# Identification of Predictors of Mood Disorder Misdiagnosis and Subsequent Help-Seeking Behavior in Individuals With Depressive Symptoms: Gradient-Boosted Tree Machine Learning Approach

**DOI:** 10.2196/50738

**Published:** 2024-01-11

**Authors:** Jiri Benacek, Nimotalai Lawal, Tommy Ong, Jakub Tomasik, Nayra A Martin-Key, Erin L Funnell, Giles Barton-Owen, Tony Olmert, Dan Cowell, Sabine Bahn

**Affiliations:** 1 Department of Chemical Engineering and Biotechnology Cambridge Centre for Neuropsychiatric Research University of Cambridge Cambridge United Kingdom; 2 Psyomics Ltd Cambridge United Kingdom

**Keywords:** misdiagnosis, help-seeking, gradient-boosted trees, machine learning, depression, bipolar disorder, diagnose, diagnosis, mood, mental health, mental disorder, mental disorders, depression, depressive, predict, predictive, prediction, depressed, algorithm, algorithms

## Abstract

**Background:**

Misdiagnosis and delayed help-seeking cause significant burden for individuals with mood disorders such as major depressive disorder and bipolar disorder. Misdiagnosis can lead to inappropriate treatment, while delayed help-seeking can result in more severe symptoms, functional impairment, and poor treatment response. Such challenges are common in individuals with major depressive disorder and bipolar disorder due to the overlap of symptoms with other mental and physical health conditions, as well as, stigma and insufficient understanding of these disorders.

**Objective:**

In this study, we aimed to identify factors that may contribute to mood disorder misdiagnosis and delayed help-seeking.

**Methods:**

Participants with current depressive symptoms were recruited online and data were collected using an extensive digital mental health questionnaire, with the World Health Organization World Mental Health Composite International Diagnostic Interview delivered via telephone. A series of predictive gradient-boosted tree algorithms were trained and validated to identify the most important predictors of misdiagnosis and subsequent help-seeking in misdiagnosed individuals.

**Results:**

The analysis included data from 924 symptomatic individuals for predicting misdiagnosis and from a subset of 379 misdiagnosed participants who provided follow-up information when predicting help-seeking. Models achieved good predictive power, with area under the receiver operating characteristic curve of 0.75 and 0.71 for misdiagnosis and help-seeking, respectively. The most predictive features with respect to misdiagnosis were high severity of depressed mood, instability of self-image, the involvement of a psychiatrist in diagnosing depression, higher age at depression diagnosis, and reckless spending. Regarding help-seeking behavior, the strongest predictors included shorter time elapsed since last speaking to a general practitioner about mental health, sleep problems disrupting daily tasks, taking antidepressant medication, and being diagnosed with depression at younger ages.

**Conclusions:**

This study provides a novel, machine learning–based approach to understand the interplay of factors that may contribute to the misdiagnosis and subsequent help-seeking in patients experiencing low mood. The present findings can inform the development of targeted interventions to improve early detection and appropriate treatment of individuals with mood disorders.

## Introduction

Mood disorders are debilitating psychiatric conditions that negatively affect a person’s emotional state. They result in impaired ability to function and complete daily tasks, and an increased risk of self-harm and suicide [1]. Two of the most common mood disorders are major depressive disorder (MDD) and bipolar disorder (BD), which affect approximately 3.4% and 0.5% of the global population, respectively, at any given time [2]. Beyond the impact on the affected individuals, there are also economic and social consequences such as lost productivity, increased health care costs, and costs incurred by unpaid carers. In the United Kingdom alone, the economic burden of managing MDD and BD is estimated at £7.5 billion (US $9.55 billion) and £5.2 billion (US $6.62 billion), respectively [3], with a significant portion of this burden attributed to underdiagnosis and high rates of misdiagnosis of mood disorders.

Although misdiagnosis is prevalent in all areas of medicine, the heterogeneous nature of mental illness and lack of objective diagnosis make it more common for mental health conditions [4]. The diagnosis of mental health disorders is currently based on assessing patient symptom profiles using diagnostic manuals such as the Diagnostic and Statistical Manual of Mental Disorders, 5th Edition (DSM-5) [5] or the International Statistical Classification of Diseases and Related Health Problems, 11th Revision (ICD-11) [6]. As such, diagnosis relies heavily on symptom reporting and patients who do not recognize and thus do not report their symptoms or present with complex symptoms are more likely to be misdiagnosed [7]. For example, issues with symptom reporting are considered a major cause of BD misdiagnosis [8], with many patients with BD only seeking medical help during depressive episodes [9], which makes mania more difficult to identify. Consequently, as many as 78% of mood disorder diagnoses are missed in primary care [10], including approximately 40% of patients with BD who are initially misdiagnosed with MDD [11]. This, in turn, leads to incorrect treatment of BD with antidepressants which have lower efficacy than mood stabilizers in alleviating bipolar symptoms and have been associated with prolonged episodes of mania and accelerated cycling between manic and depressive states [12,13]. Understanding factors that lead to misdiagnosis could guide the development of more effective means for early identification and intervention in individuals at high risk.

An additional barrier to receiving a correct diagnosis and necessary care is the reluctance of affected individuals to speak to medical professionals about their mental health. The European Study of the Epidemiology of Mental Disorders carried out across 6 countries found that only 25.4% of respondents spoke to a medical professional about their mental health problems [14]. Likewise, active engagement with mental health services is consistently low, with almost 75% of patients experiencing a mental illness in England receiving no treatment [15]. One of the reasons for the low rates of help-seeking in individuals experiencing mental health symptoms is concerns of potential public and self-internalized stigma. Consequently, individuals struggling with their mental health often turn to coping mechanisms such as social withdrawal, secrecy, and label avoidance [16,17] rather than seeking help [18]. Therefore, it is imperative to recognize barriers to help-seeking in mental health to facilitate early and accurate diagnosis in un and misdiagnosed individuals.

Although previous studies have investigated factors contributing to the misdiagnosis, poor help-seeking behavior, and barriers to receiving a diagnosis, only a few have used machine learning methods to do so [19]. The use of machine learning in mental health research has increased in recent years, with many studies focusing on detection and diagnosis, treatment and support, public health, and research and clinical administration [19]. While not without limitations, the use of machine learning can offer data-driven insights into complex relationships between high-dimensional data [20,21]. Although other, mostly qualitative investigations have identified the predictors of help-seeking and misdiagnosis by considering factors individually, this study aims to take a more holistic approach. By developing machine learning models based on extensive self-reported patient data, we aim to identify and quantify interdependent predictive factors for the misdiagnosis of mental health disorders, specifically mood disorders, and help-seeking behavior in individuals who may have been misdiagnosed. Identifying such predictive factors could aid in avoiding preventable misdiagnosis, encourage help-seeking, and improve outcomes in patients presenting with depressive symptoms.

## Methods

### Data Acquisition

#### Overview

**Figure 1:**
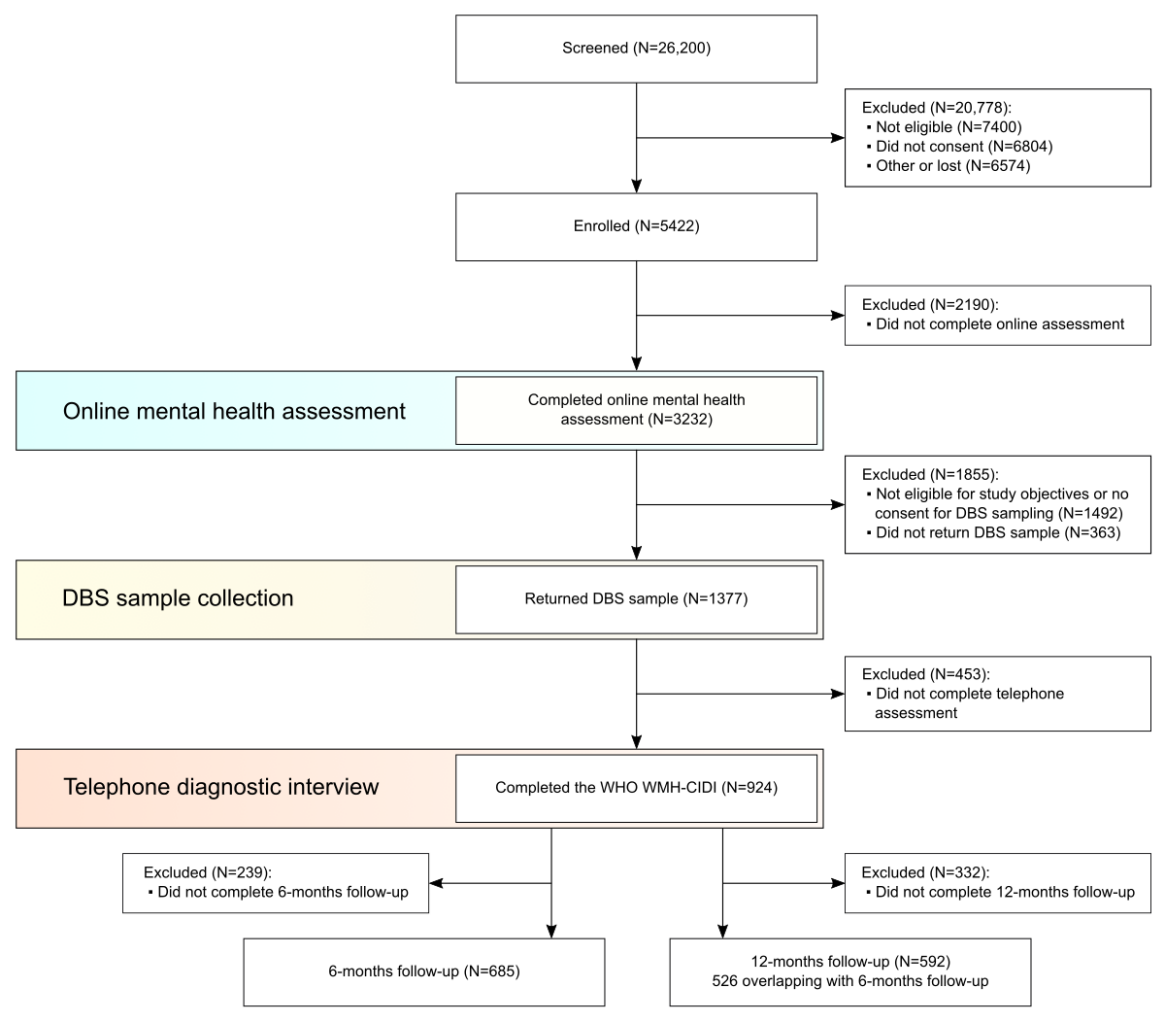
Delta Study flow diagram [22]. DBS: dried blood spot; WHO: World Health Organization; WMH-CIDI: World Mental Health Composite International Diagnostic Interview.

The data used in this report were collected as part of the Delta Study—a study aiming to facilitate a more accurate and earlier diagnosis of BD and MDD; carried out in the United Kingdom by the Cambridge Centre for Neuropsychiatric Research between 2018 and 2020 [22,23]. The study consisted of an adaptive digital questionnaire, the Composite International Diagnostic Interview (CIDI) [24], and 2 follow-up questionnaires at 6 and 12 months. The stages of the Delta Study are summarized in Figure 1. Participants were recruited nonrandomly through email, the Cambridge Centre for Neuropsychiatric Research (CCNR) website, and paid Facebook advertisements. The eligibility criteria included at least mild depressive symptoms, indicated by a score of ≥5 on the Patient Health Questionnaire-9 (PHQ-9) [25] at the time of recruitment, aged between 18 and 45 years, and residency in the United Kingdom. Participants who indicated current suicidal ideation or intent, were pregnant, or breastfeeding, were excluded.

#### Adaptive Digital Questionnaire

In total, 3232 participants completed the adaptive digital questionnaire available on the Delta Study digital platform. The questionnaire consisted of 635 questions, divided into six sections: (1) demographic information and personal history; (2) manic and hypomanic symptoms; (3) depressive symptoms; (4) personality profiling; (5) treatment, medication, substance use, and family psychiatric history; and (6) other psychiatric conditions. As the questionnaire was adaptive to answers given by participants, the maximum number of questions an individual could answer was 382, with an average of 284. Within the questionnaire, participants reported their baseline diagnosis, and their current well-being (within the previous 14 days) was quantified using the Warwick-Edinburgh Mental Well-Being Scale (WEMWBS) [26].

#### Composite International Diagnostic Interview

Participants who completed the web-based mental health questionnaire were invited to complete the CIDI version 3.0 via telephone. The CIDI is a structured diagnostic interview for mental disorders created by the World Health Organization based on the International Classification of Diseases and Related Health Problems, 10th Revision (ICD-10). It was developed primarily for epidemiological studies and has been extensively validated, demonstrating high diagnostic reliability [27]. In this study, only sections pertaining to mood disorder diagnoses were applied, that is, the demographics, depression, and mania modules. Interviewers were trained by CIDI-certified instructors prior to conducting the interviews. In total, 924 participants completed the CIDI and received one of the following diagnoses in their results report: BDI, BDII, subthreshold BD, MDD with subthreshold BD, MDD, or no mood disorder diagnosis (referred to as “low mood”).

#### Follow-Up Questionnaires

Participants who completed the digital questionnaire were invited to fill out 2 follow-up questionnaires, 6 and 12 months after receiving their results report. The follow-up questionnaires aimed to determine the effects of participation in the Delta Study on participants’ quality of life and record subsequent changes in diagnosis and treatment. A total of 2064 participants completed at least 1 of the follow-up questionnaires, with 1780 respondents at 6 months and 1542 respondents at 12 months.

#### Outcomes

##### Overview

For the purposes of this study, 2 dependent variables were defined.

##### Misdiagnosis

For participants who completed the CIDI, the mood disorder diagnosis reported at baseline was compared to the diagnosis obtained from the CIDI, including patients with no mood disorder diagnosis at baseline who should have been diagnosed. CIDI diagnosis was used as the gold standard, and any mismatch with the baseline diagnosis was defined as misdiagnosis. This definition of misdiagnosis was consistent with previous studies investigating under- and misdiagnosis of mood disorders based on comparing patient-reported diagnoses to the outcomes of structured clinical interviews [28,29].

##### Help-Seeking Behavior

In the 6- and 12-month follow-up questionnaires, participants were asked: “Have you had an appointment with a GP or psychiatrist to talk about your mental health in the past 6 months?” A positive response to this question at either time point was defined as help-seeking. In order to examine help-seeking in misdiagnosed individuals, only those who were identified as misdiagnosed within outcome 1 were included in the analysis.

### Analysis

#### Overview

Raw data processing and feature engineering were performed in R (version 3.6.3; R Core Team) [30]. Subsequent analyses and modeling were carried out using Python (version 3.9.7; Python Software Foundation) [31]. Main libraries used included Pandas (version 1.5.2; Pandas Development Team) [32] and NumPy version 1.23.5 [33] for data manipulation; scikit-learn version 1.0.2 [34], XGBoost (version 1.6.1; The XGBoost Contributors) [35], and SHAP version 0.41.0 [36] for modeling and interpretation; and Seaborn version 0.12.1 [37] and Matplotlib version 3.6.2 [38] for plotting.

#### Data Preparation

Prior to analysis, constant and duplicate variables were removed. Answers to questions examining the same symptom or construct were concatenated, and new features were created to represent these aggregated answers. Missing data were imputed where possible (for example, the answer to the question asking “Has anyone suggested you drink less?“ was set to 0 for participants who had indicated they do not drink), and otherwise remained nonrandomly missing. Categorical variables were 1-hot encoded, that is, unique dummy variables were created where the presence of each category was denoted by “1,” and its absence was represented by “0.”

#### Modeling and Interpretation

This analysis aimed to develop predictive models to identify variables influencing (1) misdiagnosis and (2) help-seeking behavior in participants who were identified as potentially misdiagnosed. A decision tree–based machine learning algorithm Extreme Gradient Boosting (XGBoost) [35] was chosen to train the classification models due to being robust to outliers, agnostic to data distribution, having the ability to handle nonrandom missing data, offering good predictive power, and due to it allowing for good model interpretability. Repeated nested cross-validation (rNCV) was used for model training and evaluation to obtain accurate estimates of model performance in unseen data. rNCV relies on performing a k-fold cross-validation (CV) within each round of another CV. This allows for model-specific hyperparameter optimization in the inner loop, with the final model being trained using the best-performing set of parameters, and later evaluated in the outer loop of rNCV. For this analysis, a 4-fold stratified CV was used in both the inner and outer loops, where 3 of the folds acted as a training set and 1 as a test set. Tuned model parameters included the number of estimators (1 to 100), shrinkage rate (0.1 to 0.3) to prevent overfitting, and tree depth (1 or 2) to allow for first-order interactions between predictors. The training was repeated 100 times, generating a total of 400 models for each of the objectives. Generalized model performance was evaluated by calculating the area under the receiver operating characteristic curve (AUC). The classification cutoff was optimized for the Youden index [39] to balance the true positive and true negative rates and offset potential imbalances between classes. SHAP (Shapley additive explanations) analysis [36], which combines local interpretable model–agnostic explanations (LIME) [40] and Shapley sampling values [41] approaches, was used for model interpretation. Feature occurrence frequency was calculated as the percentage of the models that incorporated a given feature to generate predictions. Reported results represent mean and SD values across the rNCV models.

### Ethical Considerations

The study protocol was approved by the University of Cambridge Human Biology Research Ethics Committee (HBREC 2017.11) and all enrolled participants signed a digital informed consent form.

## Results

### Misdiagnosis

The self-reported baseline diagnosis did not match the diagnosis assigned by CIDI for 471 (50.97%) of the 924 participants who completed the CIDI interview. These participants were therefore considered misdiagnosed. No between-group differences were observed in terms of age, sex, ethnicity, highest achieved education level, or relationship status between the correctly diagnosed and misdiagnosed groups (Table S1 in Multimedia Appendix 1). However, there were significant differences in employment status as well as well-being and PHQ-9 scores, with misdiagnosed individuals, on average, reporting lower well-being and more severe depressive symptoms.

**Figure 2:**
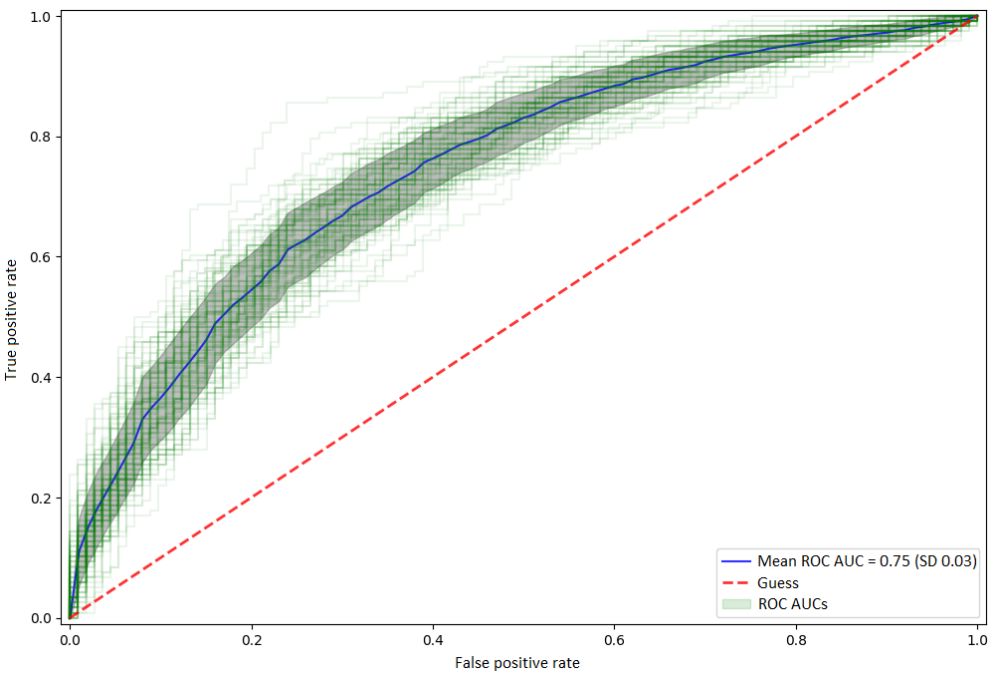
Out-of-fold model performance in predicting misdiagnosis. Green lines represent predictive performance on unseen out-of-fold data for each of the 400 final models. The thick blue line represents the average of all ROC curves. The grey area represents 1 SD. AUC: area under the receiver operating characteristic curve; ROC: receiver operating characteristic.

**Figure 3:**
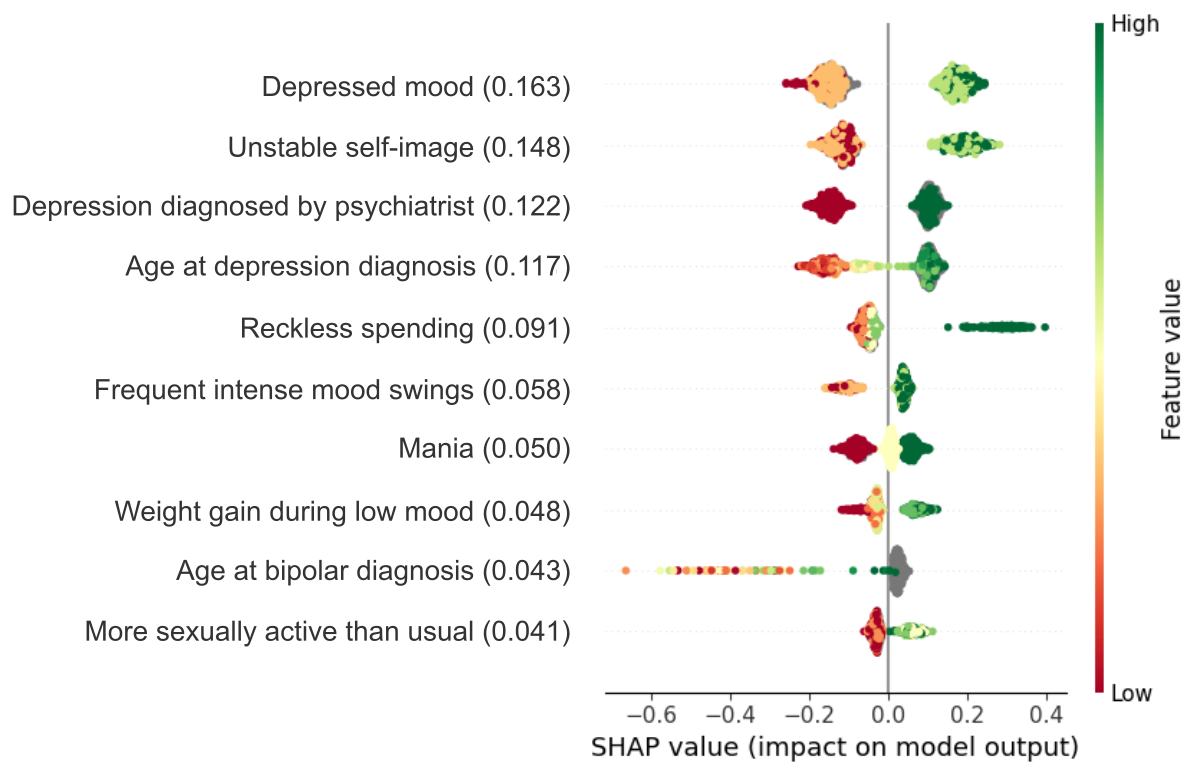
Results for the top 10 variables in the misdiagnosis model. Shown is SHAP analysis of the factors associated with misdiagnosis. The features (y-axis) are ordered by their average feature importance, indicated by the value inside the brackets, across all models. Each colored dot represents a participant, where the color gradient shows the value of the answer (red if low, green if high, and grey if missing), and the corresponding value on the x-axis shows directionality and the impact on model output, as determined using SHAP analysis. Values below 0 show directionality toward being correctly diagnosed, whereas values above 0 show directionality toward misdiagnosis. SHAP: Shapley additive explanations.

On average, the models correctly classified 70% (SD 9%) of misdiagnosed participants and 71% (SD 9%) of correctly diagnosed participants, with a mean accuracy of 70% (SD 3%) and the out-of-fold AUC of 0.75 (SD 0.03; Figure 2 and Table S2 in Multimedia Appendix 1). Among the 1045 variables evaluated, the strongest predictors of misdiagnosis were more severe composite depressive symptoms and unstable self-image (Figure 3). Unstable self-image was measured by a 4-level Likert scale question “Is your image and sense of yourself and what you believe in unstable and constantly changing?” The next strongest predictor was the diagnosing clinician, with those who were undiagnosed at baseline or reported a diagnosis by a psychiatrist more likely to be misdiagnosed. The top 10 predictors also included variables related to age at diagnosis of BD and MDD, with late (≥35 years of age) diagnosis or no diagnosis at all, increasing the likelihood of being misdiagnosed (Figure S1 in Multimedia Appendix 1). Misdiagnosed participants were also more likely to recklessly spend money, experienced more frequent intense mood swings or mania in general, had higher weight gain during low mood episodes, and were more sexually active than usual at the time of data collection.

Model performance was largely driven by the top 5 predictors, with a steady decline in SHAP scores for subsequent variables. Of the top 10 predictors, 9 were selected in more than 75% (n=300) of the models, suggesting a relatively stable model composition. The exception was a variable related to “being more sexually active than usual,” which was selected in 71% (n=284) of the models. More detailed information on feature selection frequency is provided in Figure S3 in Multimedia Appendix 1.

### Help-Seeking Behavior

Help-seeking behavior was investigated in 379 participants who were misdiagnosed at the baseline and who had completed at least 1 of the follow-up questionnaires. Of those, 229 (60.42%) participants sought an appointment with a medical professional during the follow-up period to discuss their mental health and were therefore defined as “help-seekers.” The help-seeker and non–help-seeker groups differed significantly in the highest achieved education level, relationship status, well-being, and severity of depressive symptoms (Table S3 in Multimedia Appendix 1). Participants more likely to seek help were on average less formally educated, more likely single, reported higher mean severity of symptoms, and worse overall well-being.

**Figure 4:**
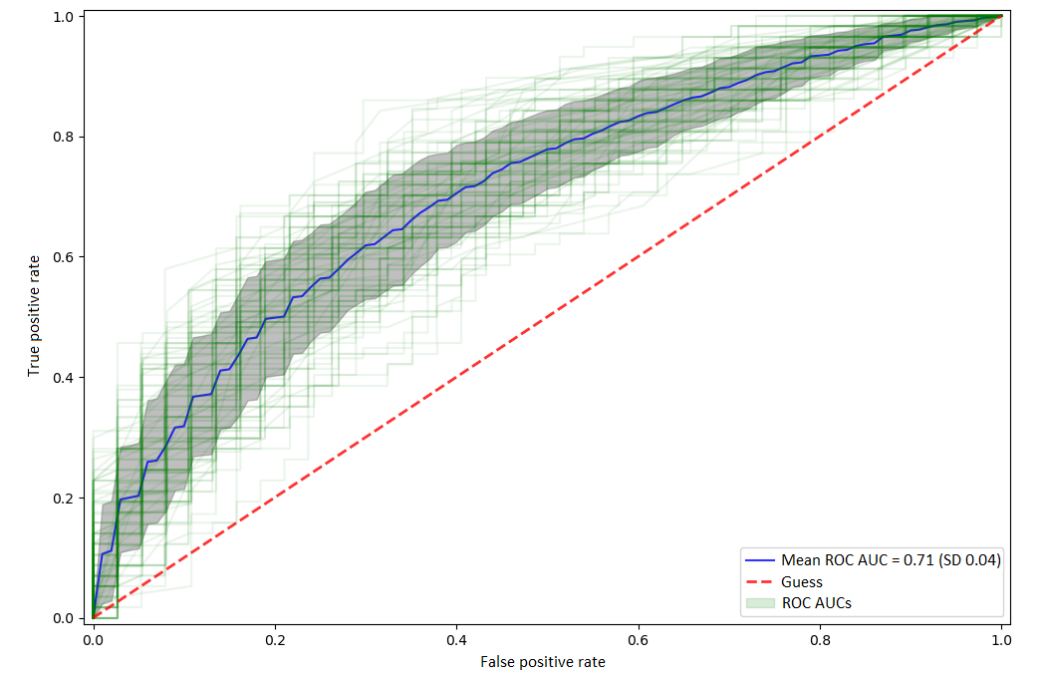
Out-of-fold model performance in predicting help-seeking. Green lines represent predictive performance on unseen out-of-fold data of each of the 400 final models. The thick blue line represents an average of all ROC curves. The grey area represents 1 SD. AUC: area under the receiver operating characteristic curve; ROC: receiver operating characteristic.

**Figure 5:**
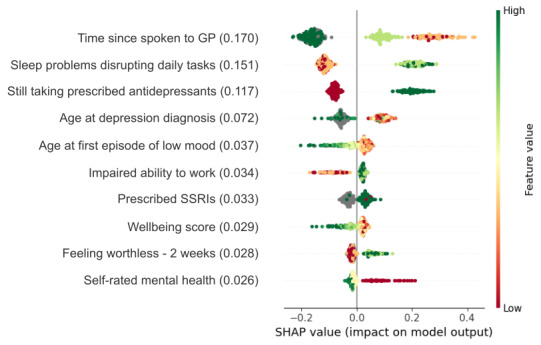
Results for top 10 variables in the help-seeking model in misdiagnosed individuals. Shown is feature SHAP importance (in brackets) and feature SHAP values (data points). SHAP values below 0 show directionality toward low help-seeking (ie, no appointment with GP or psychiatrist to discuss mental health), whereas values above 0 show directionality toward high help-seeking. GP: general practitioner; SHAP: Shapley additive explanations; SSRI: selective serotonin reuptake inhibitor.

The model achieved an AUC of 0.71 (SD 0.04; Figure 4), with a sensitivity of 65% (SD 13%), specificity of 72% (SD 13%), and average accuracy of 67% (SD 4%; Table S4 in Multimedia Appendix 1). The strongest predictor was the shorter time since patients last spoke to a general practitioner (GP) about their mental health at baseline (Figure 5). It was followed by sleep problems disrupting daily tasks and taking prescribed antidepressants, both associated with increased help-seeking. Consistent with this, lower help-seeking was observed in participants who had never been prescribed antidepressants, namely selective serotonin reuptake inhibitors (SSRIs). Furthermore, there was a lower likelihood of help-seeking with higher age at both the first episode of low mood and diagnosis of depression, which was similarly predictive to not having been previously diagnosed with depression. Finally, impaired ability to work, lower well-being scores, feeling worthless, and lower self-rated mental health were associated with help-seeking behavior.

The 3 variables, namely, time since last spoken to a GP, sleep problems disrupting daily tasks, and still taking prescribed antidepressants, were selected in nearly all models (Figure S4 in Multimedia Appendix 1), suggesting their high relevance for model predictions. Among other predictors, only age when diagnosed with depression was selected in more than 75% (n=300) of models, with the remaining features only selected in approximately 50% (n=200) of models, indicating their lower relevance.

## Discussion

### Principal Findings

This study aimed to develop machine learning models to explore factors potentially contributing to misdiagnosis and subsequent help-seeking in individuals experiencing low mood. For this purpose, we used data obtained through an extensive digital questionnaire concerning demographic, personality, and mental health data, as well as, the validated and standardized diagnostic interview, CIDI. Developed models achieved a fair level of predictive power, with AUCs of 0.75 and 0.71 for predicting misdiagnosis and help-seeking, respectively. Below, we discuss the main findings as well as the strengths and limitations of this analysis.

### Misdiagnosis

The strongest predictor of misdiagnosis was the severity of depressed mood, with more severe depressive symptoms being associated with a greater risk of being misdiagnosed. This directionality was consistent with other top predictors of misdiagnosis, including unstable self-image, reckless spending, frequent intense mood swings, mania, weight gain during low mood, and being more sexually active than usual. Except for the instability of self-image, these predictors can be divided into either depression or mania or bipolar-related symptoms. Overall, the finding that individuals with more severe mental health symptoms are at a greater risk of being misdiagnosed is surprising, given the opposite could be expected as milder symptoms are harder to detect [42]. Several factors could contribute to this association, including the complexity of diagnosing mental health disorders [43], variability in symptom presentation [44,45], and the high degree of symptom overlap across different diagnoses [5]. A possible explanation for the increased risk of misdiagnosis among individuals with more severe symptoms is that they may present with prominent mood instability, such as that observed in patients with personality disorder, or rapidly cycling symptoms, making accurate diagnosis more challenging [9]. In addition, individuals with more severe symptoms often lack motivation to seek help, hence their symptoms may remain unrecognized for a longer time [46].

In the case of mood disorders, misdiagnosis of individuals with higher depressive symptom severity may result from the fact that patients with BD generally seek medical help during depressive episodes and often present with more severe depressive symptoms than patients with MDD, while underreporting manic phases [47,48]. In fact, less than a third of patients with BD report the presence of reckless behavior, excessive spending, and increased sexual interest or activity [49]. This contributes to approximately 40% of patients with BD receiving an incorrect initial diagnosis of unipolar depression [50]. Also, the association of frequent intense mood swings with mood disorder misdiagnoses may be related to incorrect treatment of depressive symptoms of BD with antidepressants, rather than mood stabilizer medication, which has the potential to induce mania and rapid cycling [51,52].

The second most predictive feature of misdiagnosis identified in this study was unstable self-image. Previous literature has shown that an unstable sense of self is associated with frequent changes in diagnosis, and often linked to complex and unstable personality characteristics [53]. The high ranking of self-image stability could, however, be a result of the high comorbidity rates between BD and other disorders featuring unstable self-image that were not evaluated by the diagnostic interview used in this study, such as borderline personality disorder [54]. This is especially important considering that such disorders may share a high number of similarities with BD, leading to frequent misdiagnoses [55,56]. The 2 additional symptoms that are ranked high in terms of predictive value for misdiagnosis in this analysis regard reckless spending and increased sexual activity, representing reckless or impulsive behavior, which are included in the diagnostic criteria of both BD and borderline personality disorder [17].

Finally, among the top predictors of misdiagnosis were 3 variables related to psychiatric history, including psychiatrist involvement in the diagnosis, age at depression diagnosis, and age at BD diagnosis. Interestingly, the models attributed a higher risk of misdiagnosis to individuals whose depression was diagnosed by a psychiatrist. This may be caused by the fact that patients at high risk of misdiagnosis, such as those with more complex symptom presentation or suspected comorbidities, are usually referred to secondary care, following the National Institute for Health and Care Excellence (NICE) guidelines [57]. However, this finding should be interpreted with caution, as diagnoses made by psychiatrists are generally more accurate than those derived from the CIDI. Also, participants who received a diagnosis of a mood disorder at an older age, or not at all, were more likely to be misdiagnosed. This finding is surprising, as previous literature suggests that the severity and impact of symptoms decline with age, with 86% of patients with BD diagnosed by the age of 25 [58]. However, it is possible that due to milder symptoms, patients who are older may remain undiagnosed for longer periods of time.

### Help-Seeking

Analysis of participants with a mismatch between their self-reported formal diagnosis and the CIDI outcome revealed several predictors of help-seeking related to patients’ mental health history and symptoms.

The most predictive feature was time since last spoken to a GP at baseline, with patients who had visited their GP more recently being more likely to seek help. Interestingly, that was not the case for the time since last spoken to a psychiatrist, likely due to most participants not being under secondary care and the long waiting times for psychiatric assessment [59]. In line with previous literature [60], these findings indicate that help-seeking was also associated with more severe psychiatric symptoms and having a previous diagnosis of mood disorder. Similarly, participants seeking help reported lower well-being, feeling more worthless, and more functional impairment in carrying out daily tasks and at work caused by symptoms and sleep problems.

Interestingly, while there was not a significant overall age difference between the help-seekers and non–help-seekers, further analyses showed a lower tendency to seek help in individuals who were over 35 years old at initial diagnosis of depression (Figure S2 in Multimedia Appendix 1). The pattern of people who are younger being more likely to seek help is in line with the published literature [61]. Together with the finding that the initial diagnosis at older age was a strong predictor of misdiagnosis [62], this result indicates that patients who are most likely to be misdiagnosed are also the least likely to seek help. Thus, older patients may require more support to tackle potential barriers to help-seeking and receiving a diagnosis, such as stigma and inadequate mental health education [63].

The final set of predictors of help-seeking was related to previous medication. Higher help-seeking was observed in misdiagnosed individuals who were still taking previously prescribed antidepressants, in particular SSRIs, as opposed to misdiagnosed individuals who either had never been prescribed SSRIs or other antidepressant medication or had stopped taking it. The association of antidepressant treatment with help-seeking indicates that the prescribed medication may have been ineffective, as is often the case when attempting to treat depressive episodes of BD with antidepressant monotherapy [64]. Compared with the patients with MDD, the patients with BD respond worse to antidepressant medication, with short-term nonresponse rates of 51.3% in BD versus 31.6% in MDD [65]. This difference is even more pronounced in the long-term, where the loss of response to antidepressants is 3.4 times more frequent in patients with BD, while withdrawal relapse into depression is 4.7 times less frequent in BD compared to patients with MDD [65]. Moreover, individuals with unrecognized BD who are treated with antidepressants sometimes develop symptoms of mania, which in turn may motivate patients or their relatives to seek consultation with a specialist [66].

### Limitations

The main limitation of this study is the reliance on CIDI as the gold standard for mood disorder diagnosis. Although the CIDI demonstrates good agreement with structured diagnostic interviews conducted by clinicians [67], future studies should consider either retrospective or longitudinal study designs, and ideally access medical records for more accurate diagnoses, including those beyond mood disorders. Additionally, the study participants were recruited online following strict inclusion criteria and were predominantly White, necessitating further research in traditionally underrepresented ethnic minorities and more representative patient cohorts. Another limitation is the exclusion of individuals with current suicidal ideation, a characteristic that could be an important indicator of misdiagnosis. Finally, the observed associations do not necessarily imply causality, which can only be evaluated through prospective causal inference study designs.

### Conclusions

This analysis leveraged comprehensive patient data, a robust machine learning algorithm, and an extensive validation framework, to identify predictors of mood disorder misdiagnosis in individuals experiencing depressive symptoms, and subsequent help-seeking. The results highlight the increased risk for misdiagnosis associated with incomplete symptom profiles, more severe or harder to detect symptoms, and older age. Therefore, comprehensive symptom monitoring outside of depressive episodes, mental health screening at earlier ages, and clinician knowledge of the influence of advanced age on misdiagnosis risk are important considerations for early and accurate diagnosis of mood disorders. Moreover, prior engagement with mental health services, functional impairment in performing daily tasks, and younger age were associated with a higher likelihood of help-seeking. Together, these results add to the growing application of machine learning techniques in examining existing barriers to accessing mental health services [19], and may ultimately lead to the development of novel screening tools or procedures for a comprehensive mental health risk assessment in individuals presenting with mood-related symptoms.

## Appendix

Multimedia Appendix 1 Demographics, model performance metrics, dependence plots, and feature selection frequencies for all objectives.

## Abbreviations

AUC: area under the receiver operating characteristic curve
BD: bipolar disorder
CCNR: Cambridge Centre for Neuropsychiatric Research
CIDI: Composite International Diagnostic Interview
CV: cross-validation
DSM-5: Diagnostic and Statistical Manual of Mental Disorders, 5th Edition
GP: general practitioner
ICD-10: International Classification of Diseases and Related Health Problems, 10th Revision
ICD-11: International Statistical Classification of Diseases and Related Health Problems, 11th Revision
LIME: local interpretable model-agnostic explanations
MDD: major depressive disorder
NICE: National Institute for Health and Care Excellence
PHQ-9: Patient Health Questionnaire-9
rNCV: repeated nested cross-validation
SHAP: Shapley additive explanations
SSRI: selective serotonin reuptake inhibitor
WEMWBS: Warwick-Edinburgh Mental Well-Being Scale
XGBoost: Extreme Gradient Boosting
