# Supplementary material for: Identification of Predictors of Mood Disorder Misdiagnosis and Subsequent Help-Seeking Behavior in Individuals With Depressive Symptoms: Gradient-Boosted Tree Machine Learning Approach
Source: JMIR Ment Health. 2024 Jan 11;11:e50738. doi: 10.2196/50738 (PMC10811571; doi:10.2196/50738)
Supplement: Multimedia Appendix 1 [file mental_v11i1e50738_app1.docx]

### A. Demographics for all objectives

#### **A.1. Misdiagnosis**

Table S1. Demographic and clinical characteristics of study participants in the misdiagnosis group.
*GCSE = General Certificate of Secondary Education, PHQ-9 = Patient Health Questionnaire-9, SD = standard deviation, WEMWBS = Warwick-Edinburgh Mental Wellbeing Scale, MDD = Major depressive disorder, BD = Bipolar disorder, GP = general practitioner.*

|  |  | **Diagnosed Misdiagnosed**  *(n = 453) (n = 471)* | |  |  |
| --- | --- | --- | --- | --- | --- |
|  |  | **Mean (SD)** | | **U** | *p* |
| **Age (Years)** |  | 27.29 (6.98) 26.96 (6.90) | | 103143 | .382 |
|  |  | **n (%)** | | *χ*2 | *p* |
| **Sex** | Male | 155 (34.22) | 169 (35.88) |  |  |
|  | Female | 298 (65.78) | 302 (64.12) | 0.21 | .640 |
| **Education** | *<* GCSE | 5 (1.10) | 12 (2.55) |  |  |
|  | GCSE | 49 (10.82) | 55 (11.68) |  |  |
|  | A-Level | 127 (28.04) | 142 (30.15) |  |  |
|  | Undergraduate degree | 188 (41.50) | 169 (35.88) |  |  |
|  | Postgraduate degree | 84 (18.54) | 93 (19.75) | 5.19 | .270 |
| **Employment** | Employed | 240 (52.98) | 265 (56.26) |  |  |
|  | Unemployed | 47 (10.38) | 53 (11.25) |  |  |
|  | Self-employed | 27 (5.96) | 30 (6.37) |  |  |
|  | Maternity leave | 1 (0.22) | 1 (0.21) |  |  |
|  | Retired | 3 (0.66) | - |  |  |
|  | Student | 135 (29.80) | 122 (25.90) | 5.06 | .041 |
| **Ethnicity** | White  Asian / British Asian  Black / Black British  Mixed ethnicity  Others not listed  Not answered | 307 (67.77)  11 (2.43)  -  11 (2.43)  6 (1.32)  118 (26.05) | 320 (67.94)  11 (2.34)  1 (0.21)  14 (2.97)  4 (0.85)  121 (25.69) | 1.71 | .887 |

| **Relationship status** | Stable relationship | 253 (55.85) | 245 (52.02) |  |  |
| --- | --- | --- | --- | --- | --- |
|  | Unstable relationship | 33 (7.28) | 50 (10.62) |  |  |
|  | Single | 167 (36.87) | 176 (37.37) | 3.50 | .174 |
|  |  | **Mean (SD)** | | **U** | *p* |
| **WEMWBS** |  | 37.66 (8.13) 34.96 (7.65) | | 86777 | *<*.001 |
| **PHQ - 9** |  | 12.46 (4.84) 14.37 (4.95) | | 130003 | *<*.001 |
|  |  | **n (%)** | | *χ*2 | *p* |
| **Who diagnosed baseline depression** | GP | 232 (51.21) 152 (32.27) | |  |  |
|  | Psychiatrist | 53 (8.17) 58 (12.31) | |  |  |
|  | Other professional | 1 (0.22) 2 (0.24) | |  |  |
|  | Other/can’t remember | 1 (0.22) 1 (0.21) | |  |  |
|  | Not diagnosed at baseline | 181 (39.96) 258 (54.78) | | 34.48 | *<*.001 |
| **Who diagnosed baseline bipolar disorder** | GP | 1 (0.22) 1 (0.21) | |  |  |
|  | Psychiatrist | 37 (8.17) 10 (2.12) | |  |  |
|  | Other professional | 1 (0.22) 3 (0.64) | |  |  |
|  | Other/can’t remember | - 1 (0.21) | |  |  |
|  | Not diagnosed at baseline | 414 (91.39) 456 (96.82) | | 19.19 | .001 |
| **CIDI diagnosis** | MDD | 242 (53.42) 158 (53.55) | |  |  |
|  | None | 172 (37.97) 66 (14.01) | |  |  |
|  | BD | 39 (8.61) 247 (52.44) | | 215.85 | *<*.001 |

***A.2. Misdiagnosis model performance***

Table S2. Performance metrics of the model predicting misdiagnosis.

*AUC = area under the receiver operating characteristic curve, SD = standard deviation.*

| **Metric** | **Mean** | **SD** |
| --- | --- | --- |
| AUC | 0.747 | 0.027 |
| Youden’s J | 0.403 | 0.051 |
| Cutoff threshold | 0.505 | 0.052 |
| Accuracy | 0.702 | 0.026 |
| Sensitivity | 0.696 | 0.092 |
| Specificity | 0.707 | 0.092 |
| Precision | 0.718 | 0.045 |
| F1-score | 0.702 | 0.038 |

#### **A.3. Help-seeking in misdiagnosis**

Table S3. Demographic and clinical characteristics of study participants in the help-seeking in misdiagnosis group.

*GCSE = General Certificate of Secondary Education, PHQ-9 = Patient Health Questionnaire-9, SD = standard deviation, WEMWBS = Warwick-Edinburgh Mental Wellbeing Scale, MDD = Major depressive disorder, BD = Bipolar disorder, GP = general practitioner.*

|  |  | **Help-seekers Not help-seekers**  *(n = 229) (n = 150)* | |  |  |
| --- | --- | --- | --- | --- | --- |
|  |  | **Mean (SD)** | | **U** | *p* |
| **Age (Years)** |  | 26.76 (7.01) | 27.78 (7.24) | 15423 | .092 |
|  |  | **n (%)** |  | *χ*2 | *p* |
| **Sex** | Male | 74 (32.31) | 55 (36.67) |  |  |
|  | Female | 155 (67.69) | 95 (63.33) | 0.58 | .445 |
| **Education** | *<* GCSE | 4 (1.75) | 6 (4.00) |  |  |
|  | GCSE | 32 (13.97) | 14 (9.33) |  |  |
|  | A-Level | 75 (32.75) | 35 (23.33) |  |  |
|  | Undergraduate degree | 80 (34.93) | 53 (35.33) |  |  |
|  | Postgraduate degree | 38 (16.59) | 42 (28.00) | 11.71 | .019 |
| **Employment** | Employed | 126 (55.02) | 89 (59.33) |  |  |
|  | Unemployed | 28 (12.23) | 12 (8.00) |  |  |
|  | Self-employed | 15 (6.55) | 11 (7.33) |  |  |
|  | Maternity leave | 1 (0.44) | - |  |  |
|  | Retired | - | - |  |  |
|  | Student | 59 (25.76) | 38 (25.33) | 2.57 | .630 |
| **Ethnicity** | White  Asian / British Asian  Black / Black British  Mixed ethnicity  Others not listed  Not answered | 200 (87.34)  6 (2.62)  1 (0.44)  7 (3.06)  2 (0.87)  13 (5.68) | 120 (80.00)  5 (3.33)  -  7 (4.67)  2 (1.33)  16 (10.67) | 5.15 | .399 |

| **Relationship status** | Stable relationship | 107 (46.72) | 94 (62.67) |  |  |
| --- | --- | --- | --- | --- | --- |
|  | Unstable relationship | 26 (11.35) | 15 (10.00) |  |  |
|  | Single | 96 (39.20) | 41 (27.33) | 9.83 | .007 |
|  |  | **Mean (SD)** | | **U** | *p* |
| **WEMWBS** |  | 33.24 (7.02) 37.66 (7.49) | | 11545 | *<*.001 |
| **PHQ - 9** |  | 15.38 (4.84) 12.72 (4.75) | | 147500 | *<*.001 |
|  |  | **n (%)** | | *χ*2 | *p* |
| **Who diagnosed baseline depression** | GP | 90 (69.77) 31 (73.81) | |  |  |
|  | Psychiatrist | 38 (29.46) 9 (21.43) | |  |  |
|  | Other professional | - 2 (4.76) | |  |  |
|  | Other/can’t remember | 1 (0.78) - | |  |  |
|  | Not diagnosed at baseline | 100 (43.67) 108 (72.00) | | 35.02 | *<*.001 |
| **Who diagnosed baseline bipolar disorder** | GP | 1 (0.44) 1 (0.67) | |  |  |
|  | Psychiatrist | 8 (3.49) 1 (0.67) | |  |  |
|  | Other professional | 1 (0.44) 2 (1.33) | |  |  |
|  | Other/can’t remember | - 1 (0.67) | |  |  |
|  | Not diagnosed at baseline | 219 (95.63) 146 (97.33) | | 6.17 | .180 |
| **CIDI diagnosis** | MDD | 59 (25.76) 72 (48.00) | |  |  |
|  | None | 33 (14.41) 13 (8.67) | |  |  |
|  | BD | 137 (59.83) 65 (43.33) | | 20.05 | *<*.001 |

***A.4. Help-seeking model performance***

Table S4. Performance metrics of the model predicting help-seeking.

*AUC = area under the receiver operating characteristic curve, SD = standard deviation.*

| **Metric** | **Mean** | **SD** |
| --- | --- | --- |
| AUC | 0.711 | 0.044 |
| Youden’s J | 0.360 | 0.070 |
| Cutoff threshold | 0.578 | 0.078 |
| Accuracy | 0.673 | 0.043 |
| Sensitivity | 0.646 | 0.125 |
| Specificity | 0.715 | 0.126 |
| Precision | 0.785 | 0.051 |
| F1-score | 0.698 | 0.067 |

### B. Dependence plots

#### **B.1. Misdiagnosis**


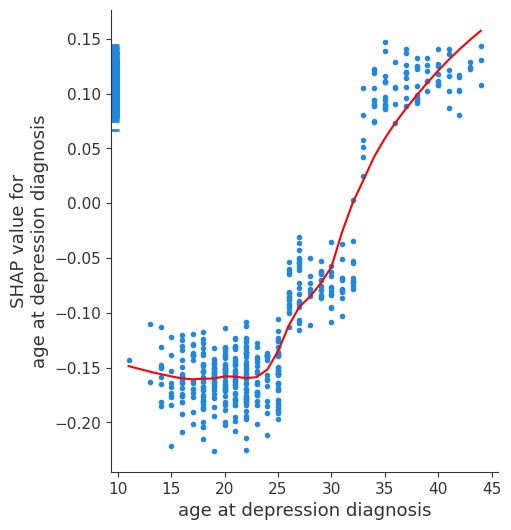

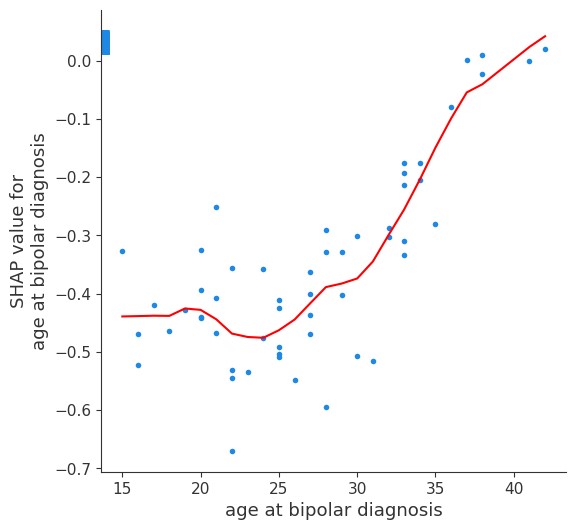


(a) Age at MDD diagnosis. (b) Age at BD diagnosis.

Figure S1. SHAP dependence plot for help-seeking in misdiagnosed individuals and (a) age at diagnosis of depression (b) PHQ-9 scores. Decreasing SHAP value represents decreasing likelihood of misdiagnosis. Points were smoothed using a local polynomial regression fit, shown in red. Missing values are represented by rectangular points.

#### **B.2. Help-seeking**


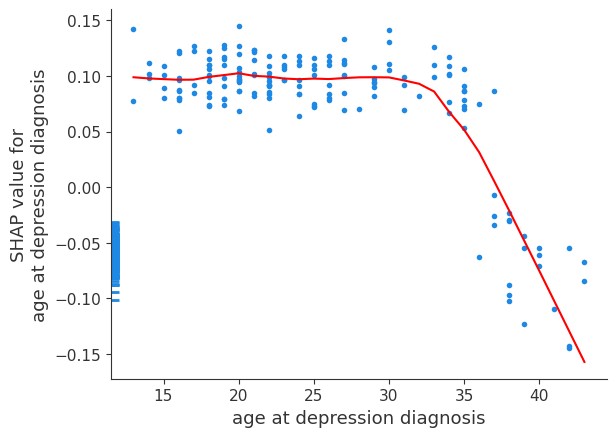

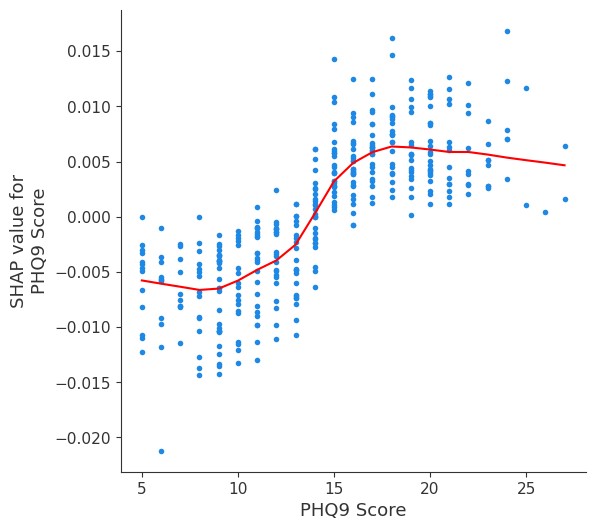


(a) Age of MDD diagnosis for help-seeking in misdiag-

nosed individuals. (b) PHQ-9 scores for help-seeking.

Figure S2. SHAP dependence plot for help seeking in misdiagnosed individuals and (a) age of diagnosis of depression (b) PHQ-9 scores. Decreasing SHAP value represents decreasing likelihood of misdiagnosis. Points were smoothed using a local polynomial regression fit, shown in red.

### C. Frequencies of variable usage for all objectives

#### **C.1. Misdiagnosis**


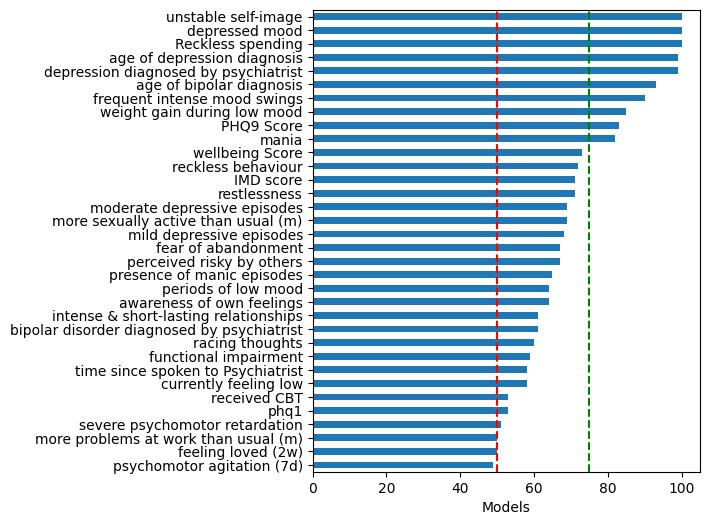


Figure S3. Feature selection frequency for the misdiagnosis models. The figure’s *y* axis shows all features available for modelling ordered by their selection frequency. Values on the *x* axis represent the number of models featuring each variable. Green line separates features used in ≥75% of all models, and red line separates features used in ≥50% of all models.

#### **C.2. Help-seeking**


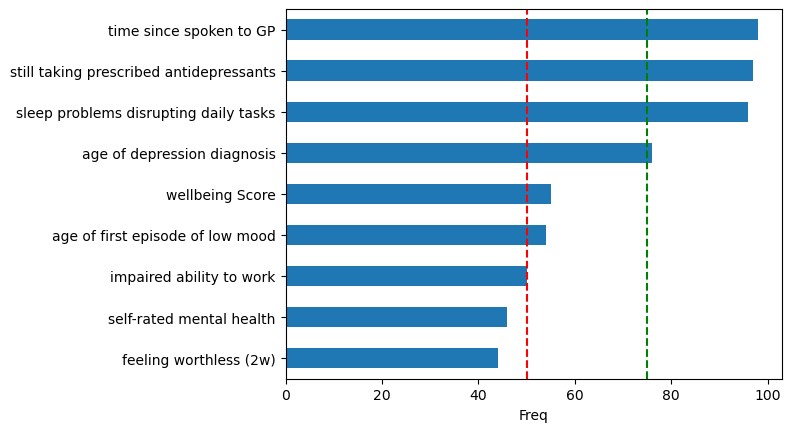


Figure S4. Feature selection frequency for the help-seeking models. The figure’s *y* axis shows all features available for modelling ordered by their selection frequency. Values on the *x* axis represent the number of models featuring each variable. Green line separates features used in ≥75% of all models, and red line separates features used in ≥50% of all models.
